# Cervical tourniquet during cesarean section to reduce bleeding in morbidly adherent placenta: a pilot study

**DOI:** 10.2144/fsoa-2021-0087

**Published:** 2022-03-08

**Authors:** Omar F Altal, Suhair Qudsieh, Abeer Ben-Sadon, Assala Hatamleh, Adel Bataineh, Omar Halalsheh, Zouhir Amarin

**Affiliations:** 1 Department of Obstetrics & Gynecology, Faculty of Medicine, Jordan University of Science & Technology, Irbid, 22110, Jordan; 2 Department of Clinical Science/Obstetrics & Gynecology, Faculty of Medicine, Yarmouk University, Irbid, 21163, Jordan; 3 Department of Anesthesia, Faculty of Medicine, Jordan University of Science & Technology, Irbid, 22110, Jordan; 4 Department of Urology, Faculty of Medicine, Jordan University of Science & Technology, Irbid, 22110, Jordan

**Keywords:** cervical ligation, cesarean section, hysterectomy, placenta accreta, uterine artery ligation

## Abstract

**Objective:**

To evaluate a modified surgical technique aiming to reduce bleeding and preserve fertility in morbidly adherent placenta by cervical tourniquet in cesarean sections.

**Methods:**

The cesarean section operations and the cervical ligation approach were performed by a single expert consultant obstetrician. The general demographics and clinical characteristics for all participants were collected and studied.

**Results:**

Eleven participants were involved. The uterus was preserved in nine patients, whereas two patients had hysterectomy. The mean blood loss was 1688.8 ml for patients whose uterus was preserved. The mean length of stay was 5.5 days.

**Conclusion:**

Cervical ligation is a simple method that can be applied by junior and experienced obstetricians to preserve the uterus.

## Plain language summary

Placenta accreta is the abnormal adhesion of placenta into the uterine wall. It carries a significant risk for hysterectomy, intrapartum and postpartum hemorrhage, and maternal morbidity and mortality. As the preservation of fertility and femininity is desired by many women, numerous methods were adapted to avoid hysterectomy in patients with placenta accreta. In this study, we describe and evaluate a novel technique to preserve the uterus by ligating the cervix with a Foley catheter.

Excessive bleeding is the leading cause of maternal death during delivery [1,2]. The definition of massive bleeding is blood loss of more than 1500 ml [3–5]. This serious complication is preventable in 90% of the cases [6]. One of the risk factors for excessive bleeding during cesarean delivery is a previous uterine scar [5,7] and morbidly adherent placenta previa [8]. Placenta accreta is the morbid adhesion of placenta into the uterine wall and abnormal invasion of trophoblasts into the myometrium [9]. It is a significant factor in maternal morbidity and mortality [10] and has a high risk of postpartum hemorrhage (PPH) [11,12]. Further, placenta accreta is a major and increasing cause of peripartum hysterectomies [13,14]. One study showed that up to one half of peripartum hysterectomies may be a result of placenta accreta [14], and another study revealed that 38% of peripartum hysterectomies are due to placenta accreta [15]. The mortality rate of placenta accreta may reach up to 7% [16]. Surgical complications also include massive transfusion, urological injury and infections [12,17]. Placenta accreta is often associated with placenta previa and previous cesarean section (CS) [18].

As the preservation of fertility is desired in most patients, the obstetrician should try all approaches to preserve the uterus in the management of placenta accreta [12]. Hysterectomy is an undesirable path to take when other measures fail to stop hemorrhage. Various management options are utilized for control of bleeding caused by this pathology, and conservative approaches are becoming increasingly used instead of hysterectomy. The aim of this study is to introduce and evaluate a technique in preservation of the uterus. This technique can be adapted by the consultant obstetrician and residents. The technique aims to ligate the internal cervical opening with a Foley catheter.

## Patients & methods

The current study was conducted at King Abdullah University Hospital affiliated with the Jordan University of Science and Technology. We prospectively identified pregnant women who were at a high risk of hysterectomy during CS as a result of the possible risk of massive bleeding in placenta accreta spectrum. The study period was 1 January to 30 June 2021. Pregnant women were offered this approach as a uterus-preserving intervention along with complete explanation of the details, including the possible complications. The following information was extracted, including age, past medical history, gravity, parity, number of previous CS, gestational age of delivery, cause of current CS, obstetrical complications, fetus status, grade of placenta previa and previous assisted reproductive techniques. In addition, data regarding the intraoperative management were collected and included type of CS incision, type of anesthesia, uterine artery ligation and other uterus-preserving methods used, hysterectomy, complications (e.g., bladder injury), estimated blood loss, and the number of packed red blood cells (PRBCs) and fresh frozen plasma transfused. Moreover, postoperative data such as postpartum complications and change in the hemoglobin level, length of hospitalization and histopathological findings were included.

The inclusion criteria was any pregnant woman with placenta accreta spectrum, including placenta accreta, placenta increta and placenta percreta, with high risk for cesarean hysterectomy and the necessity for fertility preservation. Exclusion criteria were placenta previa with no adherent capabilities in all grades, patient refusal and emergency cases without any time to offer the cervical ligation method. The American College of Obstetricians and Gynecologists guidelines were followed in risk assessment, diagnosis and management of our patients.

### Surgical settings

**Figure 1.**
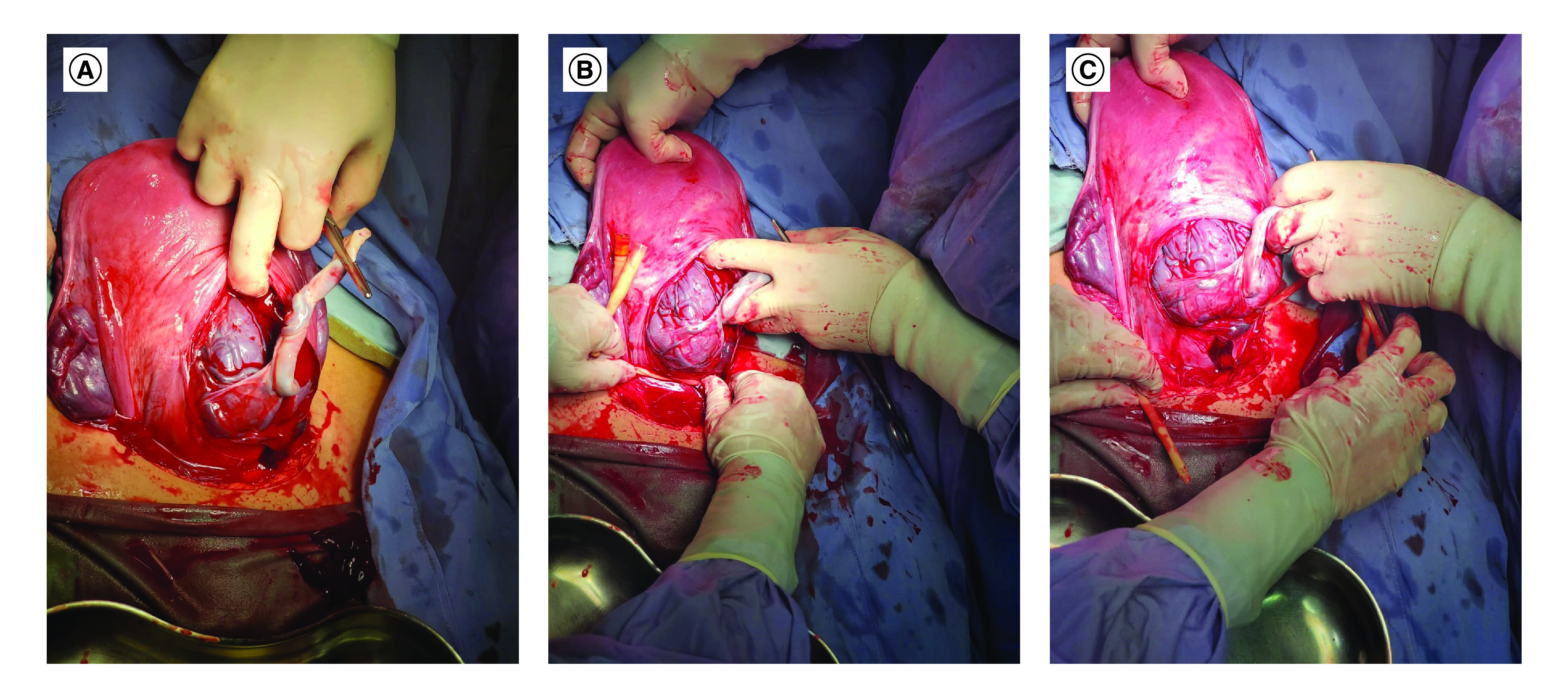
The technique of cervical ligation during cesarean section of patients with placenta accreta. **(A)** Cord clamping. **(B)** Cervical ligation by Foley's catheter. **(C)** Cessation of bleeding after cervical ligation.

Stark CS was performed in all patients included in the study. The CS operations and the cervical ligation approach were performed by a single expert consultant obstetrician. After starting the anesthesia and draping and toweling the patient, skin was opened by a low transverse incision. Then the abdominal wall was opened by layer, and the bladder was identified, dissected and pushed downward. After dissecting the vesicouterine space, the uterus was opened by a smile incision without cutting through the placenta. The baby was delivered according to the position. The placenta was not removed at this stage unless a spontaneous separation occurred. After that, the uterus was ligated with a Foley catheter at the level of the internal cervical opening to stop the supply from uterine arteries (including colpouterine vascular pedicles) in which a half hitch was made and strongly tightened up to the end. If the placenta was still adherent even with the cervical ligation, a resection in the lower uterine segment containing the strongly adherent placenta was made. Then the uterus was closed by two layers, and the Foley catheter was removed. Figure 1 & Supplementary Video 1 demonstrate the procedure.

Regarding cervical hemostatic balloon, uterine atony was prevented according to the American College of Obstetricians and Gynecologists guidelines followed at our institution. Appropriate resuscitation, uterine massage in conjunction with oxytocin, prostaglandins and ergot alkaloids were used with our surgical method. If the bleeding continued, surgical brace sutures or hysterectomy were performed. However, a hemostatic balloon was not used in any of the included cases, as the clinical setting did not necessitate its use, with good control of the bleeding.

### Statistical analysis

Data were entered into a spreadsheet. Statistical analyses were performed using IBM SPSS Statistics software, version 21 (IBM Corp., NY, USA). Limited statistical tests were applied due to the small sample size.

## Results

Eleven participants were included in the study. All pregnant women had placenta previa with ultrasonographic evidence of placenta accreta or increta. The mean age of the participants was 33.4 years (the youngest woman was 21 years and the oldest was 44 years). The gravity for all participants was 3 or more, with one of the participants having seven pregnancies. All participants had a history of one or more previous CS. The gestational age for all participants is summarized in Table 1. One participant had thrombophilia, one had systemic lupus erythematosus and one had chronic hypertension. Only one participant achieved pregnancy by *in vitro* fertilization.

**Table 1.** General demographic characteristics of the participants.

Variable	Maternal age (years)	Gravidity	Parity	Previous CS (n)	Gestational age (weeks)	Blood group	Medical illness	Assisted reproductive technique
Case 1	33	3	2	2	36 + 1	B+	No	No
Case 2	38	8	5 + 2	3	31 + 6	O+	No	No
Case 3	34	3	2	2	36 + 2	O+	No	No
Case 4	44	3	2	1	36 + 2	B+	Thrombophilia	IVF
Case 5	28	5	2 + 2	2	36 + 1	A+	No	No
Case 6	38	6	5	1	36 + 2	B+	No	No
Case 7	32	6	3 + 2	3	35	B+	No	No
Case 8	30	3	2	1	34 + 3	B+	No	No
Case 9	35	4	3	3	25 + 2	O+	SLE	No
Case 10	34	7	6	1	35 + 5	A+	HTN	No
Case 11	21	3	2	2	37 + 6	O+	No	No

CS: Cesarean section; HTN: Hypertension; IVF: *In vitro fertilization*; SLE: Systemic lupus erythematosus.

Three participants had antepartum hemorrhage. Five participants had placenta previa totalis. The details of fetus status are summarized in Table 2. During the CS, all participants had low transverse incision. With regard to hysterectomy, only in two cases (18%) was the uterus not preserved. The bladder was injured once and repaired by the consultant urologist. The mean blood loss (± standard deviation) for all participants was 2290 ml (±1589.6). For participants without hysterectomy, the mean blood loss (± standard deviation) was 1688.8 ml (±548.9). Three participants did not receive PRBC units, two received 2 units, two received 3 units, and two received 4 units. For participants who had hysterectomy, they received 6 and 8 units. Table 3 summarizes the intraoperative management and complications.

**Table 2.** The course and characteristics of the current pregnancy.

Variable	Cause of CS	Obstetric complication	Type of antepartum steroid	Fetus presentation	Femur length (weeks)	Abdominal circumference (weeks)	Birth weight (g)	Grade of PP
Case 1	2 previous CS	No	Betamethasone	Cephalic	34	35 + 5	2560	3
Case 2	3 previous CS, vaginal bleeding	APH	Betamethasone	Cephalic	31	31 + 4	1540	4
Case 3	2 previous CS	No	Betamethasone	Cephalic	34 + 2	32 + 4	2300	4
Case 4	Maternal request	No	None	Cephalic	33 + 3	34 + 1	2600	2
Case 5	2 previous CS	No	Dexamethasone	Cephalic	36 + 3	35 + 4	3100	2
Case 6	Breech presentation	No	Betamethasone	Breech	36	37 + 6	3000	3
Case 7	3 previous CS	No	Betamethasone	Cephalic	32 + 5	33 + 3	2300	4
Case 8	Vaginal bleeding; PP	APH	Betamethasone	Cephalic	32 + 2	34	2200	3
Case 9	Vaginal bleeding	APH	None	Cephalic	24 + 1	25 + 1	680	4
Case 10	Vaginal bleeding	APH	Betamethasone	Cephalic	–	–	2500	4
Case 11	2 previous CS	No	None	Cephalic	–	37 + 5	3500	3

APH: Antepartum hemorrhage; CS: Cesarean section; PP: Placenta previa.

**Table 3.** Intraoperative complications and procedure.

Variable	CS incision	Cervical opening ligation	Anesthesia	Bladder injury	Hysterectomy	Blood loss (ml)	PRBC (n)	FFP (n)
Case 1	LTCS	Yes	GA	Yes	No	1200	2	No
Case 2	LTCS	Yes	GA	No	No	2500	4	4
Case 3	LTCS	Yes	GA	No	No	1800	3	0
Case 4	LTCS	Yes	GA	No	No	2000	0	0
Case 5	LTCS	Yes	GA	No	No	1100	2	0
Case 6	LTCS	Yes	GA	No	No	1800	3	4
Case 7	LTCS	Yes	GA	Yes	Subtotal	6000	8	10
Case 8	LTCS	Yes	SA	No	No	800	0	0
Case 9	LTCS	Yes	GA	No	Total	4000	6	10
Case 10	LTCS	Yes	SA	No	No	1500	0	0
Case 11	LTCS	Yes	GA	No	No	2500	4	4

CS: Cesarean section; FFP: Fresh frozen plasma; GA: General anesthesia; LTCS: Low transverse cesarean section; PRBCs: Packed red blood cells; SA: Spinal anesthesia.

Postoperatively, patients with hysterectomy had primary post-partum hemorrhage (PPH). The mean length of stay was 5.5 days. The histopathological examination revealed placenta accreta in seven cases and placenta increta in four cases. The preoperative and postoperative hemoglobin levels are summarized in Table 4.

**Table 4.** Postoperative course of the patients.

Variable	LOS (days)	Preoperative HB (g/dl)	Postoperative HB (g/dl)	Postpartum complications	Histology findings
Case 1	4	13.4	9.5	No	Placenta accreta
Case 2	5	11.5	11.6	No	Placenta increta
Case 3	5	12.0	8.4	No	Placenta increta
Case 4	4	12.8	8.8	No	Placenta accreta
Case 5	4	10.1	8.6	No	Placenta accreta
Case 6	4	9.6	8.1	No	Placenta increta
Case 7	9	12.2	7.4	Primary PPH	Placenta increta
Case 8	4	11.4	11.1	No	Placenta accreta
Case 9	6	10.5	6.0	Primary PPH	Placenta accreta
Case 10	7	12.4	11.1	No	Placenta accreta
Case 11	5	13.4	9.5	No	Placenta accreta

HB: Hemoglobin; LOS: Length of stay; PPH: Post-partum hemorrhage.

## Discussion

To the best of our knowledge, we are the first to investigate the technique of cervical ligation with a Foley catheter during CS with placenta accreta spectrum. Eleven participants were involved in the study. The uterus was preserved in nine patients. The mean blood loss was 1688.8 ml for patients without hysterectomy. The mean length of hospitalization was 5.5 days. Many advantages of the technique can be concluded. First, the number of cesarean hysterectomies can be reduced, especially in women who require preservation of fertility. Second, the amount of blood loss and the number of transfused PRBC units are decreased. Third, this is a simple technique that can be adapted by all obstetrician levels (consultants, specialists and residents). Accordingly, in emergency cases, residents can apply the catheter around the cervix, decrease the bleeding and allow time until an experienced obstetrician arrives and the neonatologists, urologists, blood bank workers and nurses are well organized.

The incidence of placenta accreta has increased. One study in 2012 showed that the prevalence of placenta accreta was 1 in 533 cases [19]. Another study performed in 2016 revealed a rate of 1 in 272 cases of placenta accreta [20]. The increasing rate of placenta accreta is in direct relationship to the increasing CS rate, which is the most important risk factor for placenta accreta [21,22]. Other risk factors include placenta previa, advanced maternal age, increasing levels of serum α-fetoprotein and human placental lactogen, and previous uterine surgery [23–27].

The definitive treatment of placenta accreta is cesarean hysterectomy. During this operation, the placenta left *in situ* to avoid massive bleeding [28]. However, in women who have not completed their families and desire fertility, this step should be avoided and another approach or technique should be tried. One of the methods is conservative management by leaving the placenta *in situ* for spontaneous resorption and autolysis. This method includes clamping the cord close to the placenta and suturing the uterus over the placenta. Methotrexate can be given after that [29]. The average time needed for resorption of placenta is 4 weeks to 12 months, and the follow-up is done by monitoring the β-HCG level and imaging studies [30,31]. The major drawbacks for this method are the extensive need for antibiotics, approaches to minimize bleeding, and the high rate of hysterectomy [32,33].

Another technique can be done by an interventional radiologist by placing a balloon-tipped catheter with a selective embolization of the uterine vessels at the time of delivery [34,35]. There is no evidence of improvement of the outcome, and this method carries a risk of vessel thrombosis/dissection, hematoma, abscess, necrosis and pseudoaneurysm [36].

Systematic pelvic devascularization techniques involve the ligation of blood vessels that supply the uterus at different levels. The most popular technique is uterine artery ligation with an average success rate of 78.9% [37]. Bilateral uterine artery ligation can achieve a shorter operation time and less blood loss than with compression sutures [38]. Another systemic devascularization technique involves uterine and ovarian artery ligation. A study by Salvat *et al.* concluded that uterine and ovarian artery ligation achieved a 100% success rate compared with bilateral ligation of the hypogastrics with a success rate of 66% [39]. One of the levels of ligation comprises internal iliac artery ligation with a success rate of 52% [40]. A study conducted by Joshi *et al.* on the use of internal iliac artery ligation for patients with PPH showed that this technique failed to arrest hemorrhage in 33 of 84 women [41]. The study of Gungor *et al.* recommended the use of uterine and ovarian artery ligation because they found that it is more effective and technically easier than internal iliac artery ligation [40].

One of the principal techniques to stop bleeding uses compression sutures, which involves compression of the uterine corpus to stop bleeding but with different approaches according to the number of longitudinal and/or transverse sutures used, and whether or not the uterine cavity is penetrated. The success rate for uterine compression sutures ranges from 68% to 100% with an overall success rate of 92% [42].

New methods involve use of the cervix as a natural tamponade [43–45]. The technique of cervical inversion with suturing of the inverted lip of the cervix was successful in stopping bleeding in 38 of 40 patients in a study by Madny *et al.* [45]. However, they reported an abnormal cervical position in four patients after following up [45]. In our technique, we apply ligation around the cervical opening without inversion of the cervix into the uterine cavity.

In a case series of 34 women with placenta previa on the anterior uterine wall, 19 of which had accompanying placenta accreta, Kotsuji *et al.* reported the utilization of a transverse fundal incision to manually remove the placenta [48]. The procedure superiorly provides the surgeon with direct perception of the placenta after delivering the fetus, which prompts a lower bleeding volume (mean fluid loss is 1370 g including both amniotic fluid and blood) compared with the classical lower-segment CS with the aid of a modified Rubin tourniquet [46] to control the bleeding, if any occurs. In addition, none of the patients were admitted to the intensive care unit, and no maternal deaths or fetal anemia were reported, yet the length of the surgery time was longer. Nevertheless, the major concern is the risk of uterine rupture in upcoming pregnancies, which cannot be determined as this point [47]. Meng *et al.* described a two-tourniquet sequential blocking technique in instances of placenta previa accreta to control bleeding in cesarean delivery [49]. The first tourniquet was placed around the lower uterine segment’s upper region and the suspensory ligaments, whereas the second tourniquet was inserted below the round ligaments through the avascular area of the broad ligament, which was positioned below the lowest point of the placenta. Following the removal of the first tourniquet, a bilateral uterine artery ligation with interrupted circular suture or compression square suture was executed, after which the second tourniquet was removed. This procedure brought about a mean blood loss of 1286 ml; in addition, 90% of the patients (n = 18) had a successful CS, with the uterus being preserved, and postoperative favorable outcomes were reached [48]. Shmakov *et al.* compared three surgical hemostasis techniques in patients with morbidly adherent placenta, which included bilateral internal iliac artery ligation, temporary occlusion of the common internal artery with the aid of Satinsky vascular clamps, and combined compression hemostasis in which bilateral tourniquets on both the upper uterine pedicles and cervicoisthemic segment were done. Following the uterine balloon tamponade of the uterus, the invaded part of the uterus and placenta were resected. Finally, a third tourniquet was placed around the cervix. The combined compression hemostasis resulted in the lowest blood loss of all three procedures with a mean volume of 1295 ml; however, other outcomes measured did not differ among the study groups, such as hemoglobin levels, length of stay and hysterectomy rates [49]. A multicenter case series comprising 326 patients with a varying diagnosis of placenta accreta spectrum divided patients into four categories using gross invasion parameters alongside local features determined during surgery: type 1, in which the placental tissue reached the serosa, involving the upper posterior bladder; type 2, in which parametrial invasion occurred; type 3, in which the placenta invaded the low posterior bladder; and type 4, which included low posterior bladder involvement and fibrosis, correspondingly. The authors used a one-step resective conservative surgery; specifically, hemostasis was achieved using the selective ligature of vesicouterine and colpouterine vessels after the delivery of the fetus using an upper segment hysterotomy. Both the invaded tissue and placenta were removed until healthy tissue could be detected to prevent recurrence in future pregnancies. Eventually, repairing the uterus was done by employing a double-layer procedure, starting with a mattress followed by a polyglactin acid suture. The uterus could be preserved in 81.5%, 47.7%, 21.8% and 0% within types 1, 2, 3 and 4, respectively. Furthermore, one-step conservative surgery could reduce blood loss in 80% of the cases [50].

Limitations include a small sample size that should be optimized in the future to evaluate the success and possible adverse events. A long-term study assessing both radio-anatomical changes after surgery in conjunction with detailed clinical outcomes, especially with regard to adverse events in future pregnancies, and the effect of the current surgery on the incidence and management of morbidly adherent placenta diseases would be beneficial. Another limitation point is the lack of another comparing group (from another technique) to compare the outcome.

## Conclusion

In conclusion, this article introduce a modified technique to prevent hysterectomy in morbidly adherent placenta which involves ligation of cervix to stop bleeding and prevent hysterectomy. This maneuver should be useful (after a study on a large cohort of patients) in emergency cases, and residents can apply the catheter around the cervix, decreasing the bleeding and allowing time until an experienced obstetrician arrives and the neonatologists, urologists, blood bank workers and nurses are well organized. This is the core procedure, and it can achieve hemostasis alone or with another procedure such as uterine artery ligation. Further studies are needed to justify this method and other methods to preserve the uterus and femininity.

**Summary points**

- The incidence of placenta accreta and subsequent hysterectomy has increased.
- With use of a cervical tourniquet, we evaluate a modified surgical technique aiming to reduce bleeding and preserve fertility in morbidly adherent placenta.
- This technique is simple and can be applied even by residents.
